# Comparison of the evolutionary patterns of DNA repeats in ancient and young invertebrate species flocks of Lake Baikal

**DOI:** 10.18699/VJGB-23-42

**Published:** 2023-07

**Authors:** Wang Yuxiang, T.E. Peretolchina, E.V. Romanova, D.Y. Sherbakov

**Affiliations:** Limnological institute of the Siberian Branch of the Russian Academy of Sciences, Irkutsk, Russia; Limnological institute of the Siberian Branch of the Russian Academy of Sciences, Irkutsk, Russia; Limnological institute of the Siberian Branch of the Russian Academy of Sciences, Irkutsk, Russia; Limnological institute of the Siberian Branch of the Russian Academy of Sciences, Irkutsk, Russia Novosibirsk State University, Novosibirsk, RussiaIrkutsk State University, Irkutsk, Russia

**Keywords:** DNA repeats, Lake Baikal, phylogeny, Baicaliidae, amphipods, evolution of repeats, repeatome, повторы ДНК, озеро Байкал, филогения, Baicaliidae, амфиподы, эволюция повторов, репитом

## Abstract

DNA repeat composition of low coverage (0.1–0.5) genomic libraries of four amphipods species endemic to Lake Baikal (East Siberia) and four endemic gastropod species of the fam. Baicaliidae have been compared to each other. In order to do so, a neighbor joining tree was inferred for each quartet of species (amphipods and mollusks) based on the ratio of repeat classes shared in each pair of species. The topology of this tree was compared to the phylogenies inferred for the same species from the concatenated protein-coding mitochondrial nucleotide sequences. In all species analyzed, the fraction of DNA repeats involved circa half of the genome. In relatively more ancient amphipods (most recent common ancestor, MRCA, existed approximately sixty millions years ago), the most abundant were species-specific repeats, while in much younger Baicaliidae (MRCA equal to ca. three millions years) most of the DNA repeats were shared among all four species. If the presence/absence of a repeat is regarded as a separate independent trait, and the ratio of shared to total numbers of repeats in a species pair is used as the measure of distance, the topology of the NJ tree is the same as the quartet phylogeny inferred for the mitogenomes protein coding nucleotide sequences. Meanwhile, in each group of species, a substantial number of repeats were detected pointing to the possibility of non-neutral evolution or a horizontal transfer between species occupying the same biotope. These repeats were shared by non-sister groups while being absent in the sister genomes. On the other hand, in such cases some traits of ecological significance were also shared.

## Abstract (ru)

Исследованы ДНК повторы, присутствующие в геномных библиотеках с низким покрытием (0.1–0.5) четырех видов амфипод, эндемичных для озера Байкал (Восточная Сибирь), и четырех эндемичных видов брюхоногих моллюсков семейства Baicaliidae. Для этого были построены деревья методом объединения ближайших соседей для каждого квартета видов (амфиподы и моллюски) на основе соотношения повторяющихся классов, общих для каждой пары видов. Топология этих деревьев была сопоставлена с филогениями, полученными для тех же видов на основе сцепленных белок-кодирующих митохондриальных нуклеотидных последовательностей. У всех проанализированных видов в долю повторов ДНК вовлечено около половины генома. У относительно более древних амфипод (самый последний общий предок, MRCA, существовал приблизительно шестьдесят миллионов лет назад) наиболее распространенными были видоспецифичные повторы, тогда как у гораздо более молодых байкалиид (MRCA приблизительно равен трем миллионам лет) большинство повторов ДНК были общими для всех четырех видов. Если наличие/отсутствие повтора рассматривать как отдельный независимый признак, а отношение общего числа повторов в паре видов использовать в качестве меры расстояния, топология дерева NJ такая же, как и филогения квартета, выведенная для белков митогеномов, кодирующих нуклеотидные последовательности. Между тем в каждой группе видов было обнаружено значительное количество повторов, указывающих на возможность ненейтральной эволюции или горизонтального переноса между видами, занимающими один и тот же биотоп. Эти повторы были общими для неродственных групп, но отсутствовали в сестринских геномах. С другой стороны, в таких случаях некоторые черты, имеющие экологическое значение, также были общими.

## Introduction

In Metazoa, approximately half of all genomic DNA is made up of repeated DNA sequences, which are otherwise called “non-genic DNA” (Cavalier-Smith, Beaton, 1999; Bird et al., 2006) or repeatome (Titievsky et al., 2021). The already known functions of this fraction of the genome are very diverse. Most of it is satellite DNA (Biscotti et al., 2015; Silva et al., 2019; Thakur et al., 2021). A significant proportion of DNA repeats account for mobile elements belonging to different classes. There is evidence that highly repeated mobile elements may play a certain role in the regulation of genetic activity (see for example (Rocha et al., 2022)), their distribution must also be taken into account in the epigenetic analysis (Lerat et al., 2019). It is important to note that evidence is accumulating about the important role that repeated mobile elements may play in horizontal gene transfer between phylogenetically distant species (Ahmad et al., 2021; Athanasouli, Rӧdelsperger, 2022; Kejnovsky, Jedlicka, 2022). Dodsworth et al. (2015) have shown that a set of repeated elements contains a significant phylogenetic signal. They also noted the presence of a repeat fraction, which was inconsistent with the phylogenies inferred from individual nucleotide sequences and from the repeats, but treated this fraction more like an obstacle rather than an interesting phenomenon. It has been shown that horizontal gene transfer is a common mechanism of transmission of traits involved in adaptation processes in bacteria (Lee et al., 2022) and fungi (Steensels et al., 2021). Recently, there has been more and more evidence that similar mechanisms are likely to be involved in the adaptive evolution of Metazoa (Boto, 2014; Chen et al., 2017; Ahmad et al., 2021; Li et al., 2022). Sinсe then their work though the main focus of studies of the repeated DNA shifted mostly towards their potential structural role and was performed mostly of plant models (see for example (Titievsky et al., 2021)).

Here we apply the repeats analysis to the two species flocks of Baikalian invertebrates. Genetic studies of invertebrates from Lake Baikal allow to unravel many problems of their evolutionary history (Romanova et al., 2016; Peretolchina et al., 2020), mechanisms of speciation (Naumenko et al., 2017; Gurkov et al., 2019; Drozdova et al., 2022) and adaptation (Lipaeva et al., 2021), diversity and conservation (Butina et al., 2019; Yakhnenko, Itskovich, 2020). We use the features of the evolution of two species flocks of endemic Baikal invertebrates – amphipods (Bazikalova, 1945; Kamaltynov, 1999; Takhteev, 2019) and gastropods of the family Baicaliidae (Sitnikova et al., 2001; Hausdorf et al., 2003; Peretolchina et al., 2020) to study the evolution of the maximum diversity of repeats in their genomes. These two groups are attractive models for this kind of research for the following reasons: 1. Both groups of organisms have been well and comprehensively studied (see (Kozhov, 1963)). 2. Both groups evolved within Baikal, therefore all possible genome transformations were minimally, if at all, dependent on the introduction of genetic information from outside the ecosystem, and speciation processes occurred mainly by sympatric mechanisms. 3. The evolutionary histories of amphipods and Baicaliidae in Baikal are fundamentally different: if the former is represented by at least two branches that independently penetrated Baikal, the common ancestor of which existed at least 60 million years ago, then the maximum age of the common ancestor of Baikal species is at least 3 million years (Sherbakov, 1999; Mats et al., 2011).

Thus, the above-mentioned properties of evolutionary histories allow us to conduct a comparative analysis of sets of DNA repeats in two species-rich groups of invertebrates and assess the potential benefits of such a comparison for a deeper understanding of the evolutionary mechanisms that have shaped their modern diversity.

## Materials and methods

In this work, the genomic libraries of gastropods were used: Baicalia turriformis, Maackia herderiana, Korotnewia korotnewi and Godlewskia godlewskia, the collection of samples and genome-wide sequencing of which is described in Peretolchina et al. (2020). Obtaining the genomic libraries of amphipods Acanthogammarus victorii, Brachyuropus grewingkii, Garjajewia cabanisi and Macrohectopus branickii is described in (Romanova et al., 2016).

Random sets of reads were prepared from the source libraries using Seqtk-1.3 (r106) (Shen et al., 2016) on the Galaxy (Jalili et al., 2020) platform. The size of a library was set to 5 × 105 reads. The search for repeating genetic elements was performed using the RepeatExplorer (Novák et al., 2013) pipeline implemented on the Galaxy platform.

Quality control and library filtering were performed using the standard Galaxy FastQC (de Sena Brandine, Smith, 2019) tool. Cluster analysis requires files containing sequences of reads in FASTA format as input data.

The search for repeated sequences was performed using RepeatExplorer2 clustering.

Launch Parameters: Paired-end reads True, Read sampling false, Sample size 0, Select taxon and protein domain database version (REXdb) Viridiplantae version 3.0, Advanced options false, Select queue basic_fast_queue, Modify parameters (optional) –l select=1:ncpus=10:mem=32gb:scratch_local=50gb –l walltime=48:00:00 –q elixirre@pbs.elixir-czech.cz –v TAREAN_MAX_MEM=4000000,TAREAN_CPU=4

The search for repeats was limited to those that occur more often than 0.01 % of the input reads. In addition to satellite repeats, the output data contains LTR-retrotrans-posons, 45S, 5S rDNA and all other repeats, the number of which exceeds the threshold value.

Comparative analysis of the composition of repeats was performed using a set of original scripts in Python 3.10 and Biopython ver. 1.79. nblast (Costa et al., 2016) was used to compare the nucleotide sequences of DNA repeat contigs from different species.

## Results

**Fig. 1.**
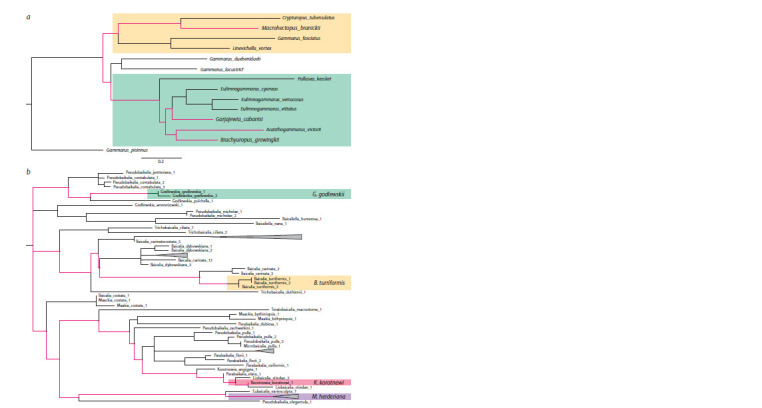
1. a, A phylogenetic tree of Baikal amphipods and some representatives of the genus Gammarus, obtained on the basis of a comparison of concatenated nucleotide sequences on which the species studied in this work are isolated. Two branches represented in Baikal are highlighted in color, as well as phylogenetic relationships between Acanthogammarus victorii, Brachyuropus grewingkii, Garjajewia cabanisi and Macrohectopus branickii. b, A phylogenetic tree of Baicaliidae, rooted at the midpoint, built on the basis of the analysis of the sequences of the Folmer fragment. The phylogenetic relationships between Baicalia turriformis, Maackia herderiana, Korotnewia korotnewi and Godlewskia godlewskia are highlighted in color.

Each of the groups of organisms is represented in this paper by four species selected in such a way that they cover the maximum range of evolutionary distances within their branch. The phylogenetic relationships of four gastropod species (Baicalia turriformis, Maackia herderiana, Korotnewia korotnewi and Godlewskia godlewskia) are shown in Fig. 1, b. The lifetime of their common ancestor does not exceed 3 million years (Zubakov et al., 1997; Sherbakov, 1999), and the connections between the selected four species pass through the root of the tree if the tree is rooted at the midpoint. It should be noted that the selected species differ dramatically in their important ecological characteristics and distribution.

Amphipods in Baikal belong to at least two large branches, both within the genus Gammarus (Sherbakov, 1999; Hou, Sket, 2016; Romanova et al., 2016; Naumenko et al., 2017). Of the species selected for the study, only M. branickii – a representative of the monotypic family – belongs to the branch ‘Micruropus’, the rest belong to the branch ‘Acanthogammarus’ – the most diverse in both species and ecology. B. grewingkii and G. cabanisi are abyssal species, A. victorii lives at shallow and medium depths, and M. branickii is a unique pelagic species distributed throughout the water column of the lake, including the maximum depths (Bazikalova, 1945) and as part of the quartet of species considered in this work is a distant outer group (see Fig. 1, a).

**Fig. 2.**
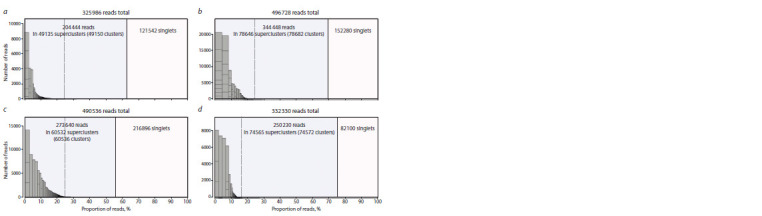
Representation of the repeats among the species of amphipods sorted by their abundance: a, Acanthogammarus victorii; b, Brachyuropus grewingkii; c, Garjajewia cabanisi, and d, Macrohectopus branickii.

Libraries of repeated contigs were constructed from genome- wide libraries of four species of amphipods and four species of gastropods of the Baikal endemic family Baicaliidae, the production of which is described in (Romanova et al., 2016) and (Peretolchina et al., 2020), respectively. 0.5 × 106 reads were randomly selected from each library and without return, resulting in depleted libraries with a coverage degree of less than 0.5, as a result of which the representation of unique sequences in them turned out to be very low. These subsets of genomic libraries were used to search and annotate DNA repeats using repeatexplorer (Novák et al., 2013). In all cases, the repetitions included approximately 50 % of the reads, which accounted for from 5 × 103 to 104 of unique contigs (Fig. 2).

The distributions of contigs by representation in genomes were also approximately the same in all cases; however, if A. victorii and G. cabanisi had a single dominant repeat (refers to simple DNA repeats, SSR) (see Fig. 2, a, c), then B. grewingkii and M. branickii had several dominant repeats (see Fig. 2, b, d ).

The comparison of the compositions of repeats in the species within each of the groups was carried out by concatenating the output files – lists of contigs resulting from a search through genomic libraries. To distinguish between contigs belonging to different species, prefix indexing was used, specific to each of the species. After converting a copy of the concatenated list into a library in blast format, we used nblast to search in the “all against all” mode. At the same time, deletions/ insertions were allowed and the similarity threshold of sequences was set to 80 %.

**Fig. 3.**
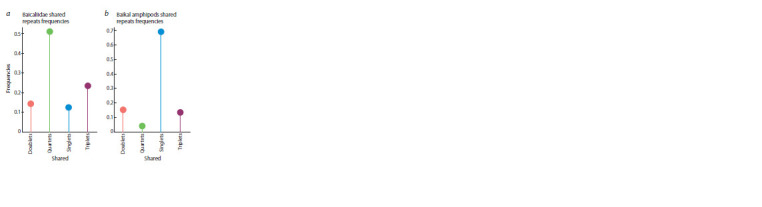
The proportions of repeats common to several species and found as a result of the blast search for gastropods Baicaliidae and amphipods

For each of the studied groups, libraries of repeat contigs were concatenated after adding species-specific tags to sequence names, then groups consisting of at least five sequences were selected and a nblast search was performed “all against all”. The search conditions allowed 20 % differences and indels. The analysis of intragroup distributions of repeats revealed significant differences between amphipods and mol- luscs (Fig. 3, see the Table). In a much younger group of Baikal, the repeats that occur in all four species turned out to be the most represented. Conversely, a relatively small proportion is accounted for by species-specific sequences (see Fig. 3, a). The representation of repetitions common to several species also turned out to be very similar in different Baikal species.

**Table 1.**
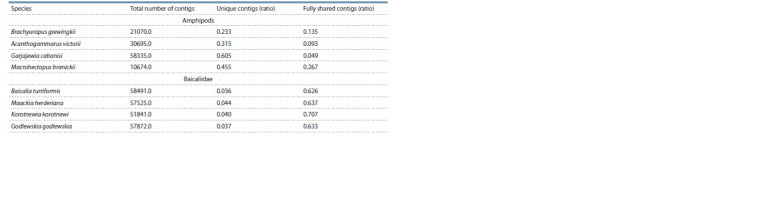
Patterns of repeats shared in amphipods and gastropods (Baicaliidae)

In amphipods, on the contrary, most of the repeats are unique (species-specific), and there are very few common ones for all four species (see Fig. 3, a). Repeats common to two, three and four species are also not equally represented in different genomes of amphipods (see the Table and Fig. 3, b). Interestingly, the largest proportion of common repeats (‘quartets’) was found in the genome of M. branickii, which is a very remote external group in relation to the other three species and, unlike the rest of the Baikal amphipods, lives in the pelagic zone of the lake.

In general, it should be noted that all possible patterns of repeat propagation are present in the genomes of both groups of species: there are both those present in only two species in all possible combinations, and all variants of absence in only one of the species (see Fig. 3).

A comparison of the distribution of repeats belonging to different classes according to their distribution in Figure 4 also does not reveal any interspecific variation in Baikal and rather significant differences between amphipod species. However, in both, all possible combinations of the two species are detected, which have repeated elements in common only for them (up to the sensitivity of detection and identification conditions). Sets of common repeats were used to cluster species. To do this, as a measure of the distance between species, we used where dij is the distance between species (genomes), i and j are species or genomes numbers, Nshared, Ni and Nj are the numbers of repeat types in the respective species. Note that the abundances of repeats of each type are not taken into account, but the denominator Ncommon involves all types of repeats found in the pulled repeats library of the species compared. These distances were used to construct the distance matrix, and it, in turn, was used to build a tree by combining the nearest neighbors (Saitou, Nei, 1987)

**Formula. 1.**
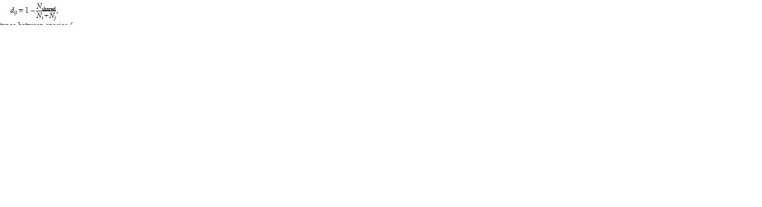
Formula. 1.

For the same species and both groups, maximum likelihood trees were inferred based on a comparison of concatenated protein-coding nucleotide sequences of mitochondrial genomes. The topologies of the trees coincided, but the ratio of nucleotide distances and distances calculated by common repetition is not linear (data not shown).

A more detailed examination of pairs of species with common repeats (Fig. 5) shows that a noticeable, albeit relatively small number of repeats is shared by species that are not sister species and thus are not consistent with the phylogeny. This property is present in both amphipods and gastropods.

## Discussion

The libraries of the NGS reads of four species of Baikal endemic gastropods (Baicalia turriformis, Maackia herderiana, Korotnewia korotnewi and Godlewskia godlewskia) belonging to the Baikal endemic Baicaliidae, and four Baikal amphipods (Acanthogammarus victorii, Brachyuropus grewingkii, Garjajewia cabanisi and Macrohectopus branickii) were used to de novo search for repeated DNA elements using the repeatexplorer algorithm. All taxa whose genomic libraries are analyzed in this work, despite various evolutionary histories, evolved within the limits of the reservoir that continuously existed on the site of modern Baikal.

The gastropods of Baicaliidae are a relatively young group, the time of the most recent common ancestors (tMRCA) of modern species is no more than 2.5 million years old. They are found at depths of no more than 100 m on a variety of soil types (Zubakov et al., 1997; Sitnikova, 2006). The amphipods in Baikal are represented by at least two large branches that diverged no earlier than about 60 million years ago (Sherbakov, 1999; Mats et al., 2011; Naumenko et al., 2017). The variety of ecological niches occupied by them is exceptionally large, they are found at all depths

By their distribution between species, all theoretically possible combinations of repeat classes were found. They ranged from species-specific ones to those found in all the genomes studied. This circumstance made it possible to use the distribution of repeats between genomes as a tool for clustering the corresponding species and comparing the topology of the obtained quartets with the results of clustering of the same species based on a comparison of the nucleotide sequences of concatenated protein-coding fragments of mitochondrial genomes. The topologies coincided, but the ratio of the lengths of the branches turned out to be different. In other words, the proportions of common repeats and the degree of differences in nucleotide sequences turned out to be independent, albeit partially correlated features

The method we used to identify highly repeated sequences and a set of search parameters allow us to identify those that are repeated in the genome with at least 50–100 copies per haploid genome. The detected repeats make up approximately 50 % of the genome and are very diverse (1× 104…6 × 104 varieties per genome, see the Table). Therefore, at the present stage of the study, we focused on the integral characteristics of this repetition and the comparison of these characteristics in two flocks of invertebrate species

The distance tree was inferred from the repeats data using the distance metric calculated from the presence/absence of a repeat class in a sample as justified by blast search under a mild set of parameters. This differs from the parsimony approach employed by (Dodsworth et al., 2015). Its advantage was in avoiding the assumption of strict homology. Nevertheless, like in their study, we obtained the same tree topology to the one inferred from mitogenome sequences for both animal groups studied. Although the topologies were the same, the ratios in branch lengths differed dramatically. We believe that these differences result from the peculiarities of the evolution of the presence/absence of repeats in genomes. The main feature is that in order to appear in the genome as a repeat, the nucleotide sequence starts as a single copy and must be amplified to such an extent that it can be detected by the repeatexplorer algorithm. The loss of repetition should also go through a gradual decrease in the number of copies of it.

Over time, differences in the compositions of repeated sequences accumulate. This confirms the spread of species-specific sequences in amphipods compared to Baicaliidae and vice versa, with a decrease in the proportion of repeats that occur in all four species. Therefore, the comparison of repeat spectra in a large number of species can be an interesting tool for phylogenetic analysis due to the high diversity of repeats and the fact that a large proportion of the genome is used in such an analysis, which gives hope for obtaining a more adequate and stable picture of evolution. A more detailed examination of pairs of species with common repeats (see Fig. 5) shows that a noticeable, albeit relatively small number of repeats, is common between species that are not sister species. This fraction, if “inconsistent”, is present in both amphipods and gastropods.

## Conclusion

Of particular interest are the repeats, the distribution of which between species contradicts the topology of phylogenetic trees, but corresponds to the ecological or geographical confinement of species. Such repeats are found in both groups (see the Table and Fig. 5), and in a significant (from hundreds to thousands) amount. From the point of view of phylogenetic analysis, they reduce its resolution but allow us to make an intriguing assumption that some part of them is involved in horizontal transfer between sympatrically inhabiting species. This requires an annotation of this part of the contigs, the results of which will be described elsewhere.

## Conflict of interest

The authors declare no conflict of interest.
